# Antioxidant and Hypolipidemic Potential of Aged Garlic Extract and Its Constituent, S-Allyl Cysteine, in Rats

**DOI:** 10.1155/2015/328545

**Published:** 2015-03-31

**Authors:** Syed Mohammed Basheeruddin Asdaq

**Affiliations:** ^1^Krupanidhi College of Pharmacy, Varthur Hobli, Bangalore 560035, India; ^2^Department of Pharmacology, Al-Maarefa College for Science and Technology, P.O. Box 71666, Riyadh 11597, Saudi Arabia

## Abstract

Aged garlic extract (AGE) is one of the unique preparations standardized with 100% bioavailable active ingredients found in the bloodstream. The current research was aimed at exploring the role of AGE and its chief active constituent, s-allyl cysteine (SAC) as antioxidant and hypolipidemic agent in rats. At the end of treatment of AGE and SAC, separated serum and freshly prepared liver tissue homogenate were analyzed for biochemical enzymes and biomarkers to evaluate and compare potencies of investigational agents. Both AGE and SAC significantly declined elevated levels of triglyceride, total cholesterol, ALP, AST, ALT, malondialdehyde, glutathione peroxidase enzyme activity, total glutathione and oxidised glutathione in serum and inclined superoxide dismutase, catalase, ferric reducing/antioxidant power, and total sulfhydryl values in liver tissue with reduction in thiobarbituric acid reactive species. The protective effects were superior with AGE compared with SAC indicating potential implication of other active constituents apart from SAC in AGE for combating hyperlipidemic stress.

## 1. Introduction

The cardiovascular morbidities are attributed to major cause of death in many parts of the world. Elevated levels of LDL (low density lipoprotein) and declined levels in HDL (high density lipoprotein) are termed as important risk factors for coronary heart disease (CHD) [1]. Currently, there is vast array of antihyperlipidemic agents for combating hyperlipidemia; however, there use is restricted due to the enhanced undesired effects they possess along with their therapeutic efficacy [2]. The preparations of plant origin is believed to be less toxic and devoid of serious side effects compared to the synthetic substances. Innumerable reports are available in favor of inverse correlation between consumption of diet rich in vegetables and fruits and occurrence of cardiovascular diseases [3–5]. Hence, it was felt worthwhile to explore role of commonly practiced herbal extract and its chief active constituent in experimental animals for obviating the hyperlipidemia and oxidative damage.

Garlic,* Allium sativum* L., and its various preparations have acquired a reputation in the traditional system over long time as effective prophylactic and therapeutic medicinal agent [6]. It has been widely acknowledged as a valuable spice and a popular remedy for different types of morbidities and disturbances in homeostasis [7]. A large number of reports demonstrate positive impact of chronic use of garlic on lowering of plasma lipids [8], systolic blood pressure [9], decrease of proinflammatory cytokines production, and reduction of platelet activation state [10]. However, chronic administration of raw garlic causes diverse toxic effects, such as anemia, weight loss, and growth reduction [11].

Aged garlic extract (AGE) is reported for less irritation and does not produce toxic changes seen with the garlic [12]. It contains sulfur compound, S-allyl cysteine (SAC) as its major constituent in addition to allin, cycloalliin, and S-allyl-L-cysteine; S-methyl-L-cysteine, S-ethylcysteine, S-1-proponyl-L-cysteine, S-allylmercapto-L-cysteine, fructosyl-arginine, and *β*-chlorogenin [13]. Further, a large number of pharmacological studies found that AGE and its components possess antioxidative, antiaging, immunomodulatory, cardiovascular, hepatoprotective [12], antiallergic [14] and cancer combating properties [15]. In consistency with the AGE, SAC is reported to be antioxidative [16, 17], anticancer [18], antihepatotoxic [19], and neurotrophic [20] and can also reduce the incidence of stroke [21].

As evident from above, both AGE and SAC possess strong potential to alleviate large number of ailments. However, there is no report on antioxidant status and antihyperlipidemic potency in comparison to each other to understand whether SAC alone is solely responsible for AGE to exhibit its lipid lowering and antioxidant property. Thus the current research was in the direction of elucidating role of SAC and AGE in combating oxidative damage and hyperlipidemia in experimental rats.

## 2. Materials and Methods

### 2.1. Preparation of AGE

Garlic bulbs were purchased during the month of December 2011 from a local market in Bangalore (India). Garlic bulbs were separated into cloves and cleansed and the skin was peeled off. The peeled cloves (400 g) were cut into small pieces and soaked in 500 mL of 25% v/v ethanol in a closed glass jar and allowed to age naturally at ambient temperature (25°C) for 10 months. The AGE was then decanted using muslin cloth [22]. Decanted extract was filtered through Whatman number 1 filter paper and the clear solution was stored at −20°C until use.

### 2.2. Materials

SAC was procured from B&K Technology Group China Co., Ltd., East Hubin Road, Xiamen, China, and required doses of SAC were prepared by using 25% v/v alcohol. All other chemicals and estimation kits were purchased from standard companies.

### 2.3. Experimental Animals

Laboratory bred female Sprague-Dawley rats weighing 220–250 g were housed at a temperature of 25° ± 5°C under 12:12 h light dark cycle. The animals were maintained under standard conditions in an animal house as per the guidelines of Committee for the Purpose of Control and Supervision on Experiments on Animals (CPCSEA). The Institutional Animal Ethics Committee approved the experimental protocol.

### 2.4. Dose Selection

The two doses of AGE (2 mL/kg and 5 mL/kg) were selected based on previous report [23]. The doses of SAC (13.1 mg/kg and 32.76 mg/kg) were selected based on HPTLC peaks of AGE as explained elsewhere by Avula et al., 2014 [24].

### 2.5. Experimental Protocol

The rats were divided into normal fat diet (NFD) and high fat diet (HFD) categories. Each category had five groups (*n* = 8). The animals in NFD category received standard rat chow (Amrut Laboratory Animal Feed, Maharashtra, India) as their feed which contained (% w/w) protein 22.10, oil 4.13, fibre 3.15, ash 5.15, and sand (silica) 1.12, whereas HFD category animals were given [25] standard rat chow, 68%; dalda (saturated fat), 30%; and cholesterol, 2% for two weeks before and during treatment.

### 2.6. Treatment

Groups I to V were fed with NFD; group I was treated with vehicle; groups II and III were given orally 2 and 5 mL/kg AGE, respectively, whereas groups IV and V were given 13.1 and 32.76 mg/kg of SAC orally. Groups VI to X were fed with HFD; group VI received vehicle; groups VII and VIII were treated with 2 and 5 mL/kg AGE orally, while groups IX and X were given SAC 13.1 and 32.76 mg/kg, p.o., respectively. All treatments were done for five consecutive days. Animal weights were recorded both at the start and before sacrificing them and percentage change in weight was calculated. Daily diet intake in grams per day for each animal was also recorded. At the end of the treatment period, the animals were fasted overnight and anaesthetized using diethyl ether. Blood was collected by puncturing retroorbital vein, centrifuged at 1000 ×g for 10 min at +4°C, and upper plasma phase was drawn with pipette and transferred into polypropylene tubes and stored at −40°C.

### 2.7. Biochemical Estimations in Serum

Determination of protein levels, estimation of glutathione peroxidase enzyme activity, and measurement of MDA levels, glutathione levels, total glutathione, and oxidized glutathione were done based on a method explained by Asdaq and Inamdar, 2010 [26]. The triglycerides (TG), total cholesterol (TC), and HDL-cholesterol (HDL) were estimated using autoanalyzer [27–29]. The LDL-cholesterol was calculated by using the following formula: [LDL in mg/dL = total cholesterol − (HDL-cholesterol − 1/5 triglycerides)] [30].

Further, atherogenic index (AI) was determined by using a method explained elsewhere [31] as AI = LDL/HDL. AST, ALT, and ALP were assayed using the corresponding commercial kits (Crest Biosystems Goa, India).

### 2.8. Biochemical Estimations in Liver Tissue Homogenate

The animals are sacrificed under deep anesthesia of diethyl ether; liver was immediately isolated and washed with normal saline and blotted with filter paper. Liver tissue homogenate (TH) was prepared in sucrose solution (0.25 M) and used for biochemical evaluation of superoxide dismutase (SOD), catalase (CAT), thiobarbituric acid reactive species (TBARS), ferric reducing/antioxidant power (FRAP), and total sulfhydryl (SH) groups assays. Measurement of thiobarbituric acid reactive species (TBARS), ferric reducing/antioxidant power (FRAP) assay, total sulfhydryl (SH) groups assay, and assay of superoxide dismutase (SOD) and catalase (CAT) were done by following a method explained by Asdaq and Inamdar, 2010 [26].

### 2.9. Statistical Analysis

Results are expressed as mean ± SEM. Significance of differences among the groups was assessed by one-way analysis of variance (ANOVA) followed by Tukey's multiple comparison tests.

## 3. Results

### 3.1. Effect of AGE and SAC on Lipid Profile

Administration of AGE and SAC (in their respective groups) to rats fed with NFD caused a significant decline in TG, TC, and LDL levels in serum with a resultant fall in atherogenic index (AI) dose dependently, whereas HDL level was significantly elevated with high dose of AGE 5 mL/kg as well as with high dose of SAC when compared to NFD control (Table 1).

When rats were fed with HFD (as described elsewhere in the paper), a significant incline in TG, TC, and LDL levels and AI was observed without any significant change in the HDL level when compared to NFD diet. Administration of AGE and SAC for five consecutive days resulted in significant reduction in TG, TC, and LDL levels as well as in AI when compared to HFD control. The high dose of AGE was found to show the best decline in lipid levels compared with high dose of SAC, while low dose of AGE showed better depletion in elevated lipid levels than low dose of SAC. Further, both the doses of AGE and SAC demonstrated a significant incline in HDL level when compared to HFD control (Table 1).

### 3.2. Effect of AGE and SAC on Serum ALP, AST, ALT, Body Weight Change (%), and Daily Diet Intake

A significant reduction in AST, ALT, and ALP levels in serum were found in rats treated with AGE and SAC (both low and high doses in their respective groups) in comparison with NFD control. Also, significant reduction in daily diet intake was observed in animals subjected to high dose of AGE and SAC without any remarkable impact on the body weight (Table 2).

Change of diet from NFD to HFD showed significant elevation in AST, ALT, ALP, and body weight with a fall in daily diet intake. Five consecutive days of treatment with AGE and SAC (in their respective groups) resulted in significant depletion in AST, ALT, ALP, and body weight with a rise in daily diet intake compared to HFD control. The high dose of AGE was superior in alleviating elevated levels of these parameters to the high dose of SAC when compared with HFD control (Table 2).

### 3.3. Effect of AGE and SAC on MDA, GSHPx, GSH, and GSSG

As shown in Table 3, administration of AGE and SAC caused a significant fall in serum MDA, GSHPx, GSH, and GSSG levels in animals fed with NFD compared to NFD control. However, introduction of HFD showed a significant rise in MDA, GSHPx, GSH, and GSSG levels when compared to NFD control. Further, five days of consecutive treatment with AGE and SAC pushes down elevated levels of MDA, GSHPx, and GSH in serum compared to HFD control. Furthermore, serum GSSG level was significantly decreased by both doses of AGE and high dose of SAC compared to HFD control.

### 3.4. Effect of AGE and SAC on SOD, CAT, TBARS, FRAP, and Total SH

Administration of high dose of AGE and SAC augmented SOD and CAT activities in liver tissue compared to NFD control. Further, FRAP and total SH values were significantly inclined in liver tissue of animals subjected to AGE (both doses) and high dose of SAC. On the other hand, both doses of AGE and high dose of SAC declined TBARS level in liver tissue compared to NFD control. HFD diet for about two weeks prior and during treatment caused significant fall in SOD, CAT, FRAP, and total SH values in liver tissue compared to NFD control. However, when rats received five consecutive days of treatment of AGE and SAC, a rise from the depleted values of SOD, CAT, FRAP, and total SH in liver tissues was observed. Further, TBARS levels were reverted back to normal values with the administration of AGE and SAC in animals (Table 4).

## 4. Discussion

The present research was aimed at elucidating the hypolipidemic potential and antioxidant status of aged garlic extract (AGE) and its major active constituent, S-allyl cysteine (SAC), in experimental models of animals. It was also my interest to find out whether SAC of AGE is solely responsible for exhibiting these therapeutic efficacy in rats subjected to HFD induced hyperlipidemia. The results of the study advocate that both AGE and SAC possess potential to alleviate hyperlipidemia induced oxidative stress with an edge in favor of AGE. These explorations indicate that there are additional constituents of AGE that might be adding therapeutic value to the SAC present in AGE and SAC alone is not responsible for therapeutic benefit of AGE in obviating hyperlipidemia and oxidative stress.

Without any further elaboration, hyperlipidemia could be termed as primary risk factor for initiation and progression of atherosclerotic lesions and subsequent cardiovascular complications. There is inverse correlation between hyperlipidemia and intact coronary function. Therefore, lowering elevated serum level of cholesterol is the cornerstone therapy for amelioration of coronary heart disease. In the current study, hyperlipidemia was induced with high fat diet consisting of dalda (30%), a saturated fat, and pure cholesterol (2%) in normal diet. Animals were kept on this diet two weeks prior to administration of intended drugs, that is, AGE and SAC. A rise in TG, LDL, and TC level was observed without altering HDL level. AGE and SAC treatment decreased the serum TG levels. This inclination could be attributed to AGE and SAC mediated increased release of endothelium bound lipoprotein lipase, which hydrolyses TG into fatty acids [32]. High level of TC and more specifically LDL-cholesterol are major coronary risk factors [33]. LDL is attributed to deposition of cholesterol in the arteries and aorta culminating in coronary heart diseases [34]. Administration of both high and low doses of AGE and high dose of SAC remarkably depletes both TC and LDL indicating cardioprotective potential of AGE and SAC. Elevated level of cardioprotective lipoprotein HDL after treatment with higher doses of AGE and SAC in HFD group could be due to increase in activity of lecithin-cholesterol acyl transferase (LCAT), which contributes to regulation of blood lipids. LCAT plays a key role in incorporating free cholesterol into HDL and transferring back to VLDL and IDL, which is taken back by liver cells [35]. Several studies show that an increase in HDL is associated with decrease in coronary risk [36] and most of the drugs that decrease TC also decrease HDL. On the contrary, in the current research, both AGE and SAC decreased the TC and LDL but enhanced HDL substantially indicating its merit over other hypolipidemics. AGE and SAC mediated hypolipidemic potential has been due to deactivating 3-hydroxy-3-methylglutaryl-CoA by as much as 41% [37].

Development of hyperlipidemia also results in damage to hepatocyte membrane leading to leakage of endogenous enzymes into circulation [38, 39]. Hence the protection offered by AGE and SAC was evaluated based on the level of enzymes such as AST, ALT, and ALP leaked into the serum from liver. Both AGE and SAC substantially restored normal level of enzymes in the serum proposing protection offered to hepatocytes by effectively lowering lipid level.

It is well known that the stress due to hyperlipidemic leads to the release of oxidative free radicals. A small rise in hydrogen peroxide tends to cause inactivation of catalase and glutathione peroxidase despite normal mRNA expression [40, 41]. Moreover, we also observed increased MDA and GSHPx activity levels in plasma of HFD fed animals. Catalase and glutathione peroxidase catalyze the decomposition of hydrogen peroxide. We know that concentration of H_2_O_2_ is high in areas of damage, and the diffusion of H_2_O_2_ into plasma will be relatively more limited and significantly high. GSHPx activities will be able to lead to the detoxification of H_2_O_2_ easily in the plasma. One of the sources of plasma antioxidant enzymes is lysis of erythrocytes. Increased plasma GSHPx activities may be the result of lysis of erythrocytes due to decreased GSHPx activity and increased ROS levels in erythrocytes. The consequence of increased free radicals via H_2_O_2_ generation and imbalances in oxidant/antioxidant balance is oxidative stress, which leads to oxidative damage, resulting in increased MDA levels, which is the end product of lipid peroxidation [42]. In the current study, we found that both AGE and SAC prevented the elevation of MDA, GSHPx, GSH, and GSSG in serum resulting in potent antioxidant effect.

Under abnormal physiological condition such as hyperlipidemia, the balance between oxidant and antioxidant system is bound to get altered [43, 44]. Hence we evaluated the antioxidant/reducing potential of liver tissue muscle using FRAP assay which appears with a significant fall in antioxidant power, as indicated by FRAP value, in HFD control group. Both AGE and SAC restored FRAP value to almost normal level. Sulfhydryl (SH) groups play an important role in biochemical and metabolic activities in the body such as cell division, blood coagulation, maintenance of protein systems, and enzymatic activation including antioxidant enzymes (catalase, superoxide dismutase, etc.) [45]. Sulfhydryl (SH) groups are also known to be sensitive to oxidative damage and falls during stress such as hyperlipidemia. AGE and SAC exhibited higher SH contents in liver tissues than their HFD control, indicating their help in replenishing the total thiol pool. Higher concentrations of AGE were more effective than their SAC counterpart in showing high levels of total SH group as well as FRAP values.

As evident from above, both AGE and SAC are effective substances in alleviating oxidative damage and hyperlipidemia. However, extent of protection offered by high dose of AGE is higher than the high dose of SAC. Being a principal/major constituent of AGE, SAC demonstrates therapeutic advantages, but this does not mean that SAC is the only constituent responsible for AGE's pharmacological impact. This is confirmed by enhanced therapeutic benefit of high dose of AGE compared with the high dose of SAC.

## 5. Conclusion

Both AGE and its active constituent, SAC, were effective in obviating hyperlipidemia and scavenging oxidative free radicals induced by high fat diet in experimental animals. My interesting finding is the enhanced therapeutic benefit of AGE compared with its active constituent, SAC, in exhibiting therapeutic benefits. These suggest that, apart from SAC of AGE, there are other constituents responsible for synergistic antihyperlipidemic and antioxidant potential of AGE.

## Figures and Tables

**Table 1:** Effect of AGE and SAC on serum triglyceride (TG), total cholesterol (TC), HDL, LDL, and LDL/HDL ratio (AI).

Treatment	TG (mg/dL)	TC (mg/dL)	HDL (mg/dL)	LDL (mg/dL)	AI
NFD control	71.04 ± 0.51	79.39 ± 0.81	33.10 ± 0.23	60.49 ± 0.55	1.82 ± 0.12
NFD + AGE-L	63.40 ± 1.26^∗∗^	65.12 ± 1.49^∗∗∗^	41.23 ± 1.28^∗^	36.57 ± 0.87^∗∗∗^	0.88 ± 0.07^∗∗∗^
NFD + AGE-H	50.12 ± 0.93^∗∗∗^	55.64 ± 0.33^∗∗∗^	44.38 ± 0.96^∗∗∗^	21.28 ± 1.07^∗∗∗^	0.47 ± 0.02^∗∗∗^
NFD + SAC-L	65.39 ± 1.30^∗∗^	68.21 ± 0.74^∗∗∗^	39.49 ± 1.24	41.79 ± 0.91^∗∗∗^	1.05 ± 0.21^∗∗∗^
NFD + SAC-H	61.61 ± 0.21^∗∗∗^	61.21 ± 1.09^∗∗∗^	42.52 ± 1.41^∗∗∗^	31.01 ± 1.16^∗∗∗^	0.72 ± 0.06^∗∗∗^
HFD control	118.23 ± 1.18^aaa^	123.33 ± 0.71^aaa^	31.07 ± 0.81	115.90 ± 0.82^aaa^	3.73 ± 0.33^aaa^
HFD + AGE-L	91.20 ± 1.01^∗∗∗^	94.14 ± 1.44^∗∗∗^	46.22 ± 1.65^∗∗∗^	66.16 ± 0.97^∗∗∗^	1.43 ± 0.21^∗∗∗^
HFD + AGE-H	79.32 ± 1.09^∗∗∗^	81.23 ± 1.66^∗∗∗^	53.89 ± 0.89^∗∗∗^	43.20 ± 0.65^∗∗∗^	0.80 ± 0.05^∗∗∗^
HFD + SAC-L	99.12 ± 1.43^∗∗∗^	101.40 ± 1.06^∗∗∗^	43.24 ± 0.50^∗∗^	77.98 ± 0.48^∗∗∗^	1.80 ± 0.36^∗∗∗^
HFD + SAC-H	81.322 ± 0.68^∗∗∗^	96.38 ± 0.98^∗∗∗^	48.89 ± 1.45^∗∗∗^	63.75 ± 0.43^∗∗∗^	1.30 ± 0.21^∗∗∗^

Values are expressed as mean ± SEM of eight rats. AGE-L: low dose of AGE, 2 mL/kg; AGE-H: high dose of AGE, 5 mL/kg; SAC-L: low dose of SAC, 13.1 mg/kg; and SAC-H: high dose of SAC, 32.76 mg/kg.

Symbols represent statistical significance.

^∗∗∗^ *P* < 0.001; ^∗∗^ *P* < 0.01 and ^∗^ *P* < 0.05 NFD fed versus NFD control and HFD fed versus HFD control respectively. ^aaa^ *P* < 0.001 NFD control versus HFD control.

**Table 2:** Effect of AGE and SAC in serum ALP, AST, ALT, body weight change (%), and daily diet intake.

Treatment	ALP (IU/L)	AST (IU/L)	ALT (IU/L)	Body weight (%)	Diet intake (g/day)
NFD control	199.4 ± 11.1	24.1 ± 1.5	17.2 ± 1.1	11.2 ± 1.2	15.1 ± 1.1
NFD + AGE-L	174.3 ± 10.2^∗∗^	15.2 ± 1.4^∗∗∗^	14.9 ± 1.2^∗∗∗^	11.2 ± 1.2	15.9 ± 1.9
NFD + AGE-H	151.4 ± 9.9^∗∗∗^	13.2 ± 1.9^∗∗∗^	12.1 ± 0.9^∗∗∗^	12.8 ± 1.2	14.6 ± 1.2
NFD + SAC-L	189.3 ± 8.2^∗^	20.1 ± 1.3^∗∗^	15.9 ± 0.4^∗^	11.8 ± 1.1	16.2 ± 1.2
NFD + SAC-H	164.3 ± 8.4^∗∗∗^	16.1 ± 1.1^∗∗∗^	13.9 ± 0.6^∗∗∗^	12.7 ± 1.2^∗∗∗^	15.1 ± 1.1^∗^
HFD control	316.2 ± 9.3^aaa^	42.7 ± 1.2^aaa^	39.2 ± 1.1^aaa^	25.5 ± 1.9^aaa^	11.7 ± 1.8^aaa^
HFD + AGE-L	249.4 ± 4.6^∗∗∗^	32.6 ± 1.2^∗∗∗^	32.1 ± 2.2^∗∗∗^	18.3 ± 1.0^∗∗^	13.6 ± 1.2^∗^
HFD + AGE-H	211.3 ± 10.7^∗∗∗^	26.2 ± 1.3^∗∗∗^	23.9 ± 1.5^∗∗∗^	12.9 ± 1.9^∗∗∗^	16.5 ± 1.4^∗∗∗^
HFD + SAC-L	269.2 ± 10.5^∗∗^	36.9 ± 1.2^∗∗^	35.1 ± 1.3^∗∗^	20.2 ± 1.2^∗^	12.9 ± 1.2^∗^
HFD + SAC-H	227.3 ± 11.7^∗∗∗^	31.2 ± 1.3^∗∗∗^	28.8 ± 1.2^∗∗∗^	14.4 ± 1.9^∗∗∗^	14.5 ± 1.5^∗∗∗^

Values are expressed as mean ± SEM of eight rats. AGE-L: low dose of AGE, 2 mL/kg; AGE-H: high dose of AGE, 5 mL/kg; SAC-L: low dose of SAC, 13.1 mg/kg; and SAC-H: high dose of SAC, 32.76 mg/kg.

Symbols represent statistical significance.

^∗∗∗^ *P* < 0.001; ^∗∗^ *P* < 0.01 and ^∗^ *P* < 0.05 NFD fed versus NFD control and HFD fed versus HFD control respectively. ^aaa^ *P* < 0.001 NFD control versus HFD control.

**Table 3:** Effect of AGE and SAC on MDA, GSHPx, GSH, and GSSG.

Treatment	MDA (nmol/mL)	GSHPx (U/mg protein)	GSH (nmol/mL)	GSSG (nmol/mL)
NFD control	0.48 ± 0.02	0.46 ± 0.03	5.09 ± 0.02	4.54 × 10^−2^ ± 0.3 × 10^−2^
NFD + AGE-L	0.37 ± 0.01^∗∗^	0.39 ± 0.02^∗∗∗^	4.08 ± 0.37^∗∗^	3.93 × 10^−2^ ± 0.3 × 10^−2∗^
NFD + AGE-H	0.28 ± 0.02^∗∗∗^	0.29 ± 0.05^∗∗∗^	3.81 ± 0.22^∗∗∗^	2.98 × 10^−2^ ± 0.4 × 10^−2∗∗∗^
NFD + SAC-L	0.41 ± 0.04^∗^	0.41 ± 0.02^∗∗^	4.78 ± 0.24^∗^	4.10 × 10^−2^ ± 0.5 × 10^−2∗^
NFD + SAC-H	0.33 ± 0.03^∗∗^	0.31 ± 0.03^∗∗∗^	4.15 ± 0.38^∗∗^	3.28 × 10^−2^ ± 0.6 × 10^−2∗∗∗^
HFD control	0.81 ± 0.09^aaa^	0.71 ± 0.04^aaa^	6.87 ± 0.27^aaa^	6.88 × 10^−2^ ± 0.2 × 10^−2aaa^
HFD + AGE-L	0.62 ± 0.01^∗∗∗^	0.48 ± 0.08^∗∗^	5.59 ± 0.29^∗∗^	5.02 × 10^−2^ ± 0.6 × 10^−2∗∗^
HFD + AGE-H	0.41 ± 0.09^∗∗∗^	0.33 ± 0.04^∗∗∗^	4.60 ± 0.54^∗∗∗^	3.97 × 10^−2^ ± 0.3 × 10^−2∗∗∗^
HFD + SAC-L	0.63 ± 0.05^∗∗^	0.58 ± 0.06^∗∗^	5.984 ± 0.51^∗∗^	5.99 × 10^−2^ ± 0.3 × 10^−2^
HFD + SAC-H	0.47 ± 0.10^∗∗∗^	0.39 ± 0.05^∗∗∗^	5.13 ± 0.16^∗∗^	4.82 × 10^−2^ ± 0.1 × 10^−2∗∗∗^

Values are expressed as mean ± SEM of eight rats. AGE-L: low dose of AGE, 2 mL/kg; AGE-H: high dose of AGE, 5 mL/kg; SAC-L: low dose of SAC, 13.1 mg/kg; and SAC-H: high dose of SAC, 32.76 mg/kg.

Symbols represent statistical significance.

^∗∗∗^ *P* < 0.001; ^∗∗^ *P* < 0.01 and ^∗^ *P* < 0.05 NFD fed versus NFD control and HFD fed versus HFD control respectively. ^aaa^ *P* < 0.001 NFD control versus HFD control.

**Table 4:** Effect of AGE and SAC on SOD, CAT, TBARS, FRAP, and total SH groups assay.

Treatment	SOD (unit/g tissue)	CAT (unit/g tissue)	TBARS (nmol/g tissue)	FRAP value (*µ*mol/g tissue)	Total sulfhydryl (*µ*mol/g tissue)
NFD control	78.5 ± 1.8	77.3 ± 1.5	32.91 ± 1.3	3.93 ± 0.6	0.69 ± 0.04
NFD + AGE-L	84.1 ± 1.2	83.6 ± 2.4^∗^	28.11 ± 1.4^∗^	4.89 ± 0.5^∗^	0.78 ± 0.08^∗^
NFD + AGE-H	92.4 ± 1.4^∗^	97.2 ± 1.9^∗^	22.43 ± 2.1^∗∗∗^	5.69 ± 0.4^∗∗∗^	0.86 ± 0.02^∗∗^
NFD + SAC-L	81.1 ± 1.2	87.9 ± 1.9^∗^	29.43 ± 2.1	4.10 ± 0.1^∗^	0.71 ± 0.01^∗^
NFD + SAC-H	89.5 ± 1.1^∗^	93.0 ± 1.1^∗^	24.22 ± 1.7^∗∗^	5.11 ± 0.4^∗∗∗^	0.81 ± 0.03^∗∗^
HFD control	41.2 ± 1.3^aaa^	40.1 ± 1.6^aaa^	47.21 ± 1.1^aaa^	2.91 ± 0.7^aaa^	0.31 ± 0.05^aaa^
HFD + AGE-L	62.0 ± 1.6^∗∗∗^	68.7 ± 1.5^∗∗^	35.89 ± 1.9^∗∗∗^	3.96 ± 0.4^∗∗∗^	0.45 ± 0.03^∗^
HFD + AGE-H	73.1 ± 1.3^∗∗∗^	80.4 ± 1.9^∗∗∗^	24.79 ± 1.6^∗∗∗^	4.23 ± 0.3^∗∗∗^	0.61 ± 0.02^∗∗∗^
HFD + SAC-L	58.2 ± 1.5^∗∗^	59.3 ± 1.1^∗∗^	38.88 ± 1.7^∗∗^	3.83 ± 0.2^∗^	0.38 ± 0.03^∗^
HFD + SAC-H	70.1 ± 1.3^∗∗∗^	71.2 ± 1.2^∗∗∗^	29.66 ± 1.4^∗∗∗^	4.02 ± 0.6^∗∗∗^	0.58 ± 0.01^∗∗∗^

Values are expressed as mean ± SEM of eight rats. AGE-L: low dose of AGE, 2 mL/kg; AGE-H: high dose of AGE, 5 mL/kg; SAC-L: low dose of SAC, 13.1 mg/kg; and SAC-H: high dose of SAC, 32.76 mg/kg.

Symbols represent statistical significance.

^∗∗∗^ *P* < 0.001; ^∗∗^ *P* < 0.01 and ^∗^ *P* < 0.05 NFD fed versus NFD control and HFD fed versus HFD control respectively. ^aaa^ *P* < 0.001 NFD control versus HFD control.
